# Selenium deficiency-induced alterations in ion profiles in chicken muscle

**DOI:** 10.1371/journal.pone.0184186

**Published:** 2017-09-06

**Authors:** Haidong Yao, Xia Zhao, Ruifeng Fan, Hamid Sattar, Jinxin Zhao, Wenchao Zhao, Ziwei Zhang, Yufeng Li, Shiwen Xu

**Affiliations:** 1 Department of Veterinary Medicine, Northeast Agricultural University, Harbin, P. R. China; 2 CAS Key Laboratory for Biomedical Effects of Nanomaterials and Nanosafety, and Center for Environmental Safety and Health, Institute of High Energy Physics, Chinese Academy of Sciences, Beijing, China; Auburn University, UNITED STATES

## Abstract

Ion homeostasis plays important roles in development of metabolic diseases. In the present study, we examined the contents and distributions of 25 ions in chicken muscles following treatment with selenium (Se) deficiency for 25 days. The results revealed that in chicken muscles, the top ranked microelements were silicon (Si), iron (Fe), zinc (Zn), aluminum (Al), copper (Cu) and boron (B), showing low contents that varied from 292.89 ppb to 100.27 ppm. After Se deficiency treatment, essential microelements [Cu, chromium (Cr), vanadium (V) and manganese (Mn)], and toxic microelements [cadmium (Cd) and mercury (Hg)] became more concentrated (P < 0.05). Elements distribution images showed generalized accumulation of barium (Ba), cobalt (Co), Cu, Fe and V, while Cr, Mn, and Zn showed pin point accumulations in muscle sections. Thus, the ion profiles were generally influenced by Se deficiency, which suggested a possible role of Se deficiency in muscle dysfunctions caused by these altered ion profiles.

## 1. Introduction

Selenium (Se) is known to play crucial roles in many aspects of human and animal health [1, 2]. Se is an essential component of different types of enzymes and antioxidants, such as glutathione peroxidase, and other selenoproteins [3, 4]. Numerous previous studies have shown that Se involves in the process of muscle metabolism [4], redox regulation [5], neurobiology process [6], and regulation of selenoproteins [7, 8]. However, Se deficiency has been associated with cardiac and skeletal muscle diseases [9] such as white muscle disease in sheep, Keshan disease in human [10]. Se deficiency either induce typical clinical and pathological changes, or cause various pathological responses at molecular levels [4]. In chicken, Se deficiency induces the occurrence of exudative diathesis, and nutritional muscular dystrophy, so muscle is one important target organ of Se deficiency [7]. Oxidative and inflammatory injury and apoptosis are reported to involve in Se deficiency induced injury [11, 12], but the possible mechanism still remains elusive.

Ion homeostasis plays important roles in the global epidemic trend of nutritional and metabolic diseases and toxonosis in human and animals [13]. The disordered iron (Fe), calcium (Ca), magnesium (Mg) and zinc (Zn) are linked with hyperglycemia and type 2 diabetes [13], and cardiovascular disease [14]. In addition, toxic elements (aluminium (Al), arsenic (As), cadmium (Cd), lead (Pb), mercury (Hg), etc.) accumulation has also been linked to serious diseases and developmental disorders, such as, reproductive impairment, immune-system diseases, neurological disorders as well as carcinogenic effects [15]. These reports show that ion contents have close relation with the development of types of diseases, so it is worth doing to understand the trait of ion profiles in a specific disease or injury.

Se could interact with other toxic ions in plants and animals [16–18], indicating that Se may serve as an antagonist to counteract the toxicity of metals. In contrary, Se deficiency also influence the levels of Cd in rat [19], Hg in fish [20], and Ca in chicken muscle [21]. However, it is still unclear about the initial molecular mechanism of this type of disorder. In chicken, trace elements distribution and concentration have already been described in different development stages of chicken [22]. However, whether the ion homeostasis is influenced by Se deficiency is unclear. Therefore, we conducted the present study to determine: 1) the ion profiles following the treatment of Se deficiency; 2) detect the relationship between different ions and analyze the role of ions in the process of Se deficiency.

## 2. Material and methods

### 2.1 Birds and diets

All procedures used in this study were approved by the Institutional Animal Care and Use Committee of Northeast Agricultural University [8]. 180 male broiler chickens (1 day old; Weiwei Co. Ltd., Harbin, China) were randomly divided into two groups (90 chickens per group). Over the entire experimental period (25 days), the chickens were allowed ad labium consumption of feed and water. The chickens were maintained either on a Se-deficient diet (-Se group) containing 0.008 mg Se/kg or on Se-adequate diet (supplemented with sodium selenite) (Control group) containing 0.2 mg Se/kg. Each group, 90 chickens were separated into 6 pens (15 chickens each pen). Chickens were euthanized at 25 days old (when they get sick and showed typical clinical symptom: depression and cannot move normally in the cage, exudative diathesis under skin, bleeding under skin and muscle). Following euthanasia with sodium pentobarbital, the pectoral muscles were removed. The tissues were rinsed with ice-cold sterile de-ionized water, frozen immediately in liquid nitrogen, and stored at -80°C until required. Some parts of muscle tissue specimens were rapidly fixed in 10% neutral-buffered formalin solution for elemental imaging.

### 2.2 Mineral element analysis

The mineral elements, lithium (Li), boron (B), sodium (Na), Mg, aluminum (Al), silicium (Si), potassium (K), vanadium (V), chromium (Cr), Mn, Fe, cobalt (Co), nickel (Ni), copper (Cu), Zn, arsenic (As), Se, molybdenum (Mo), Cd, stannum (Sn), stibium (Sb), barium (Ba), Hg, thallium (Tl) and Pb, in pectoral muscles were determined using inductively coupled plasma mass spectrometry ICP-MS (ThermoiCAPQ, American). The instrumental parameters of the equipment used are summarized in **S1 Table**.

The mineral element concentrations were determined in acid digested samples according to the method described by [22]. One gram of each sample was digested with 5 mL nitric acid (HNO_3_) (65%) and 2 mL H_2_O_2_ (30%) in microwave digestion system and diluted into 10 mL with de-ionized water. A blank digest was carried out in the same way. Digestion conditions for microwave system were applied as 3 min for 1800 W at 100 ^o^C, 10 min for 1800 W at 150 ^o^C and 45 min for 1800 W at 180 ^o^C. The digested samples were filled with ultrapure water to 10 mL before analysis by ICP-MS.

### 2.3 Elemental imaging in muscle tissues with SRμ-XRF

The SRμ-XRF technique is a powerful tool for non-destructive elemental analysis with exceptional sensitivity. To reveal the influence of Se deficiency on muscles, 2D elemental distribution of the microelements, were imaged using synchrotron radiation micro X-ray fluorescence (SRμ-XRF). Muscle tissue specimens were rapidly fixed in 10% neutral-buffered formalin solution for at least 24 h. The fixed specimens were dehydrated through a graded series of ethanol (70%, 80%, 95%, 95%, 100%, and 100%) for 1–2 h at each concentration, cleared in xylene for 15 min, embedded in paraffin, and then cut into 5-mm-thick sections. The slices were fixed onto 1 mm-thick glass slide, and then analyzed by SRμ**-**XRF according to the method by Li [23].

Elemental distribution was imaged using SRμ-XRF and analyzed according to [18]. The storage ring ran at an energy of 3.5 GeV with a current intensity of 200–300 mA. An excitation energy of 13 keV was chosen to excite the elements. The SR beamline was monochromatized with a Si (111) double-crystal monochromator and focused to 5 × 5 μm^2^ with a K-B system for analysis of the muscle slices. The samples were fixed on a moving platform and moved along the horizontal × vertical direction using stepped motors and the pixel step size was set to 5 μm for muscle slices scanning, with a dwell time of 2 seconds.

### 2.4 Statistical analysis

Statistical analysis of the obtained data was performed using Statistical Product and Service Solutions (SPSS) for Windows (version 13, SPSS Inc., Chicago, IL). The differences between the Se deficiency group and the control group were assessed by using paired t-test. The data were expressed as the mean ± standard deviation. Differences were considered to be significant at P < 0.05. In addition, principal component analysis (PCA) was used to define the most important parameters that could be used as key factors for individual variations.

The fluorescence intensities of the elements were recorded and analyzed using element detector combined with a multiple channel analyzer. The counts of the elements were normalized to that of the I0 to correct the effect of the SR beam flux variation on the signal intensity, and then imaged using OriginPro. 9.0 software. The normalized X-ray fluorescence intensities are scaled from blue (minimum) to red (maximum). These images visually demonstrate the distributions and accumulations of microelements.

## 3. Results

### 3.1 Effect of Se deficiency on macroelements in chicken muscle

**Fig 1:**
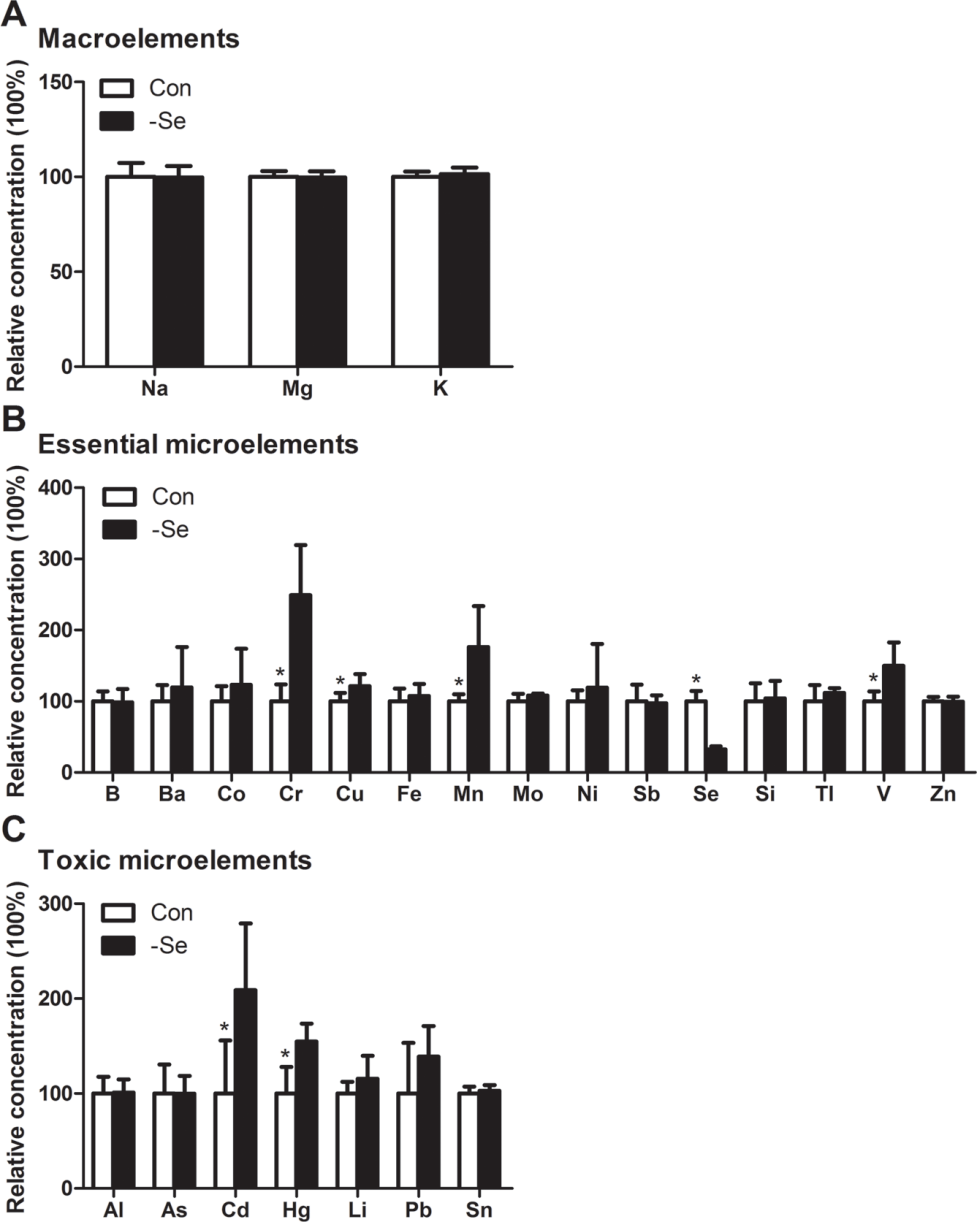
The ion profiles in chicken muscles detected by ICP-MS. A. the contents of macroelements; B. the contents of essential microelements; C. the contents of toxic microelements.* Significant difference from the corresponding control (P < 0.05). The data are expressed as the means ± SD, *n* = 6.

**Fig 2:**
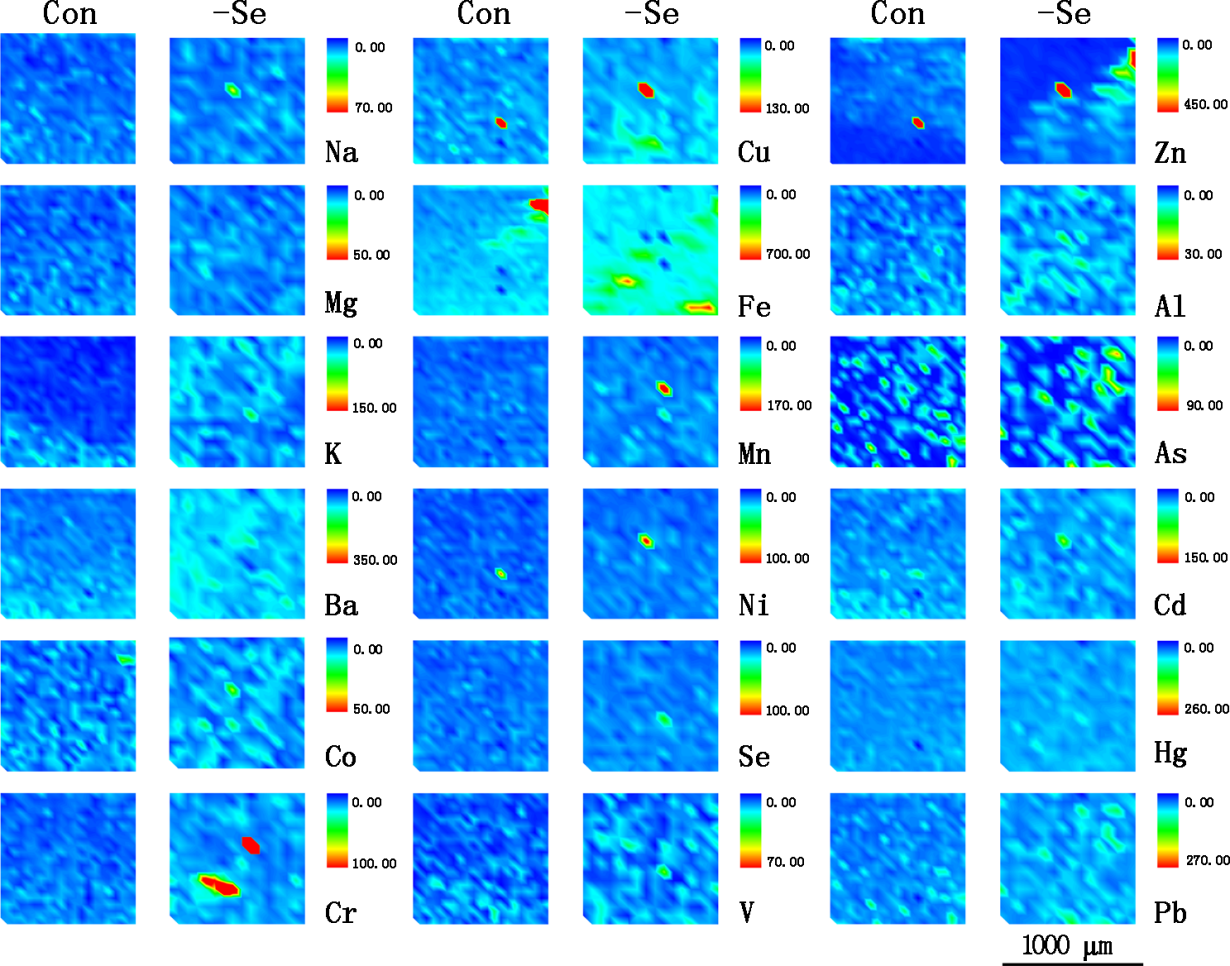
The distributions of ion profiles in chicken muscles. 5-mm-thick muscles sections were detected by SRμ-XRF.

In the present study, we examined macroelements including Na, Mg, and K in chicken muscle by ICP-MS. The results (**Fig 1A**) showed that the contents of K were the highest followed by Na and Mg. Following Se deficiency treatment, the contents of the macroelements were not significantly influenced (P > 0.05). Similar to the results detected by ICP-MS, the elements distribution (**Fig 2**) examined by SRμ-XRF also showed that the distribution of K, Na and Mg were visually not influenced. In addition, we found that Se deficiency decreased Ca content in chicken muscle in our previous study [21]. The results showed that the effect of Se deficiency on the levels of macroelements was not significant.

### 3.2 Effect of Se deficiency on essential microelements in chicken muscle

We examined the contents of essential microelements such as B, Ba, Co, Cr, Cu, Fe, Mn, Mo, Ni, Se, Si, Sb, Tl, V and Zn by ICP-MS. The results (**Fig 1B**) showed that among these detected microelements, Cr, Cu, Mn, and V were significantly increased by Se deficiency, but Se was decreased (P < 0.05). The distribution of essential microelements was examined by SRμ-XRF (**Fig 2**). However, only Ba, Co, Cr, Cu, Fe, Mn, Ni, Se, V and Zn were detected in the present study. The distribution images showed that Ba, Co, Cu, Fe and V were generally accumulated in Se-deficient muscle sections, but Cr, Mn, and Zn were focused on small points in Se-deficient muscle sections, however, the distributions of Se and Ni were not visually significant.

### 3.3 Effect of Se deficiency on toxic microelements in chicken muscle

In the present study, we detected 7 toxic microelements, Al, As, Cd, Hg, Li, Pb and Sn, using ICP-MS. The results showed that Se deficiency significantly increased the contents of Cd and Hg (P < 0.05) (**Fig 1C**), but not other elements (P > 0.05). The images of toxic microelements detected by SRμ-XRF (**Fig 2**) showed that Al, As and Pb were visually accumulated in muscle sections. Although the difference was not significant in the distribution images of Cd and Hg, the signal was also higher in the Se deficiency groups than the control. Furthermore the distributions of these microelements were generally consistent with the elements contents detected by ICP-MS.

### 3.4 Principal component analysis

**Fig 3:**
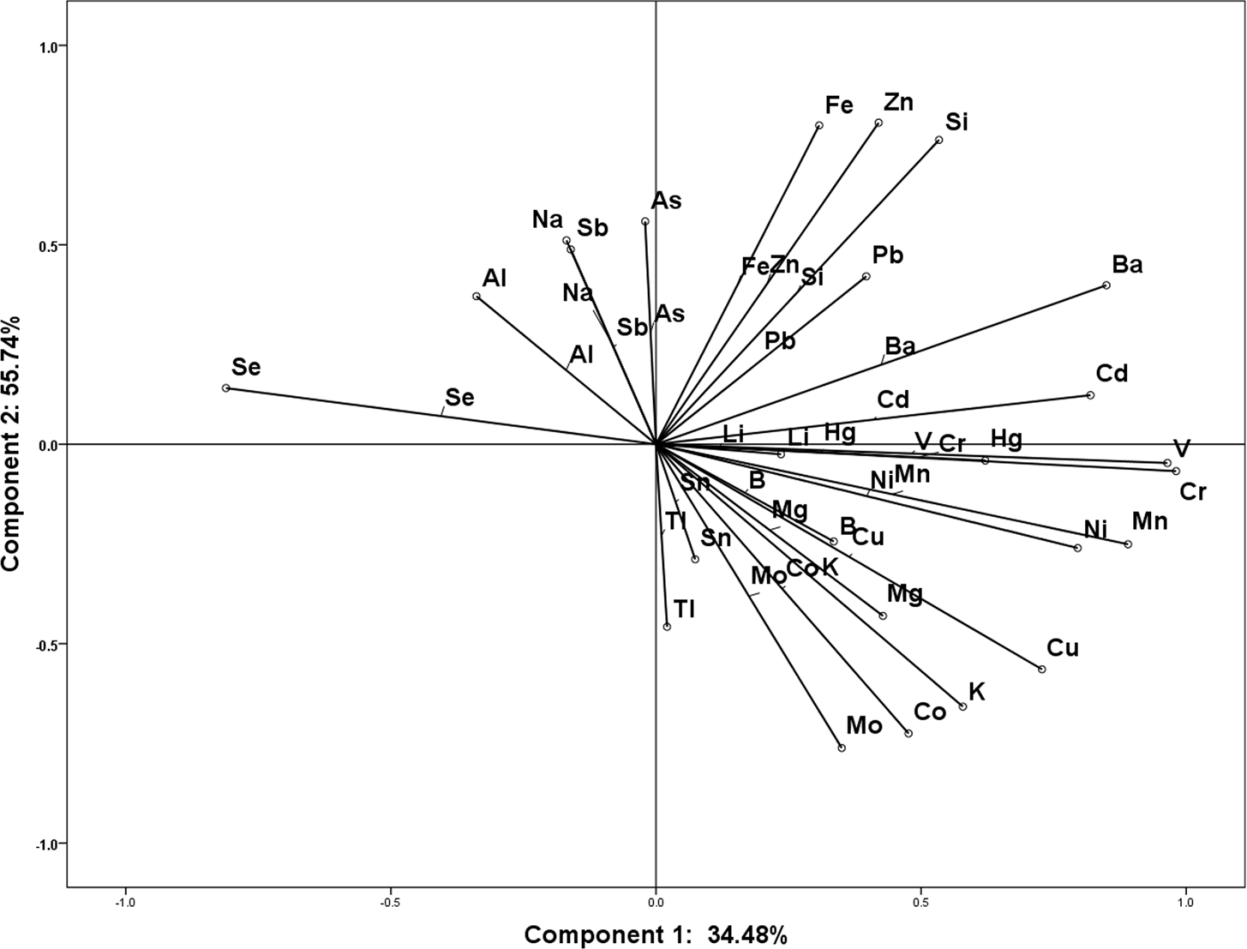
Ordination diagram of the principal component analysis (PCA) of parameters measured in chicken muscles.

In the present study, we further analyzed the interaction between these elements by using PCA (**Table 1**). All parameters were distinguished on ordination plots corresponding to the first and second principal components (34.48, and 55.74%, respectively) (**Fig 3**). The correlation between different ions was confirmed and quantified according to Pearson correlation test. The results showed both positive and negative correlations between different ions. B, Mg, Si, As and Sb showed positive correlation with Se, however other elements showed negative correlations. In addition, Na, Al, and Sb showed positive correlation with component 2, but negative correlation with component 1, which was similar to Se. Li, Si, Cr, Fe, Zn, As, Cd, Ba, Hg and Pb showed positive correlation with both component 1 and 2. The results showed that the correlation between ions in chicken muscle was complex, and in response to Se deficiency these elements could be divided into several groups.

**Table 1:** Rotated component matrix^a^.

Components	Li	B	Na	Mg	Al	Si	K	V	Cr	Mn	Fe	Co	Ni	Cu	Zn	As	Se	Mo	Cd	Sn	Sb	Ba	Hg	Tl	Pb
**Component 1**	0.03	0.04	-0.01	0.04	-0.03	0.09	0.06	0.12	0.12	0.11	0.06	0.04	0.09	0.08	0.07	0.01	-0.10	0.03	0.11	0.00	-0.01	0.12	0.08	-0.01	0.06
**Component 2**	0.00	-0.04	0.09	-0.07	0.06	0.15	-0.10	0.02	0.01	-0.02	0.15	-0.12	-0.03	-0.08	0.15	0.10	0.01	-0.13	0.04	-0.05	0.08	0.09	0.01	-0.08	0.09

^a^ Extraction Method: Principal Component Analysis. Rotation Method: Varimax with Kaiser Normalization. Rotation converged in 3 iterations.

## 4. Discussion

Ions are involve in broad range of important biological processes, and are closely related to human and animal health. In the present study, we detected 25 ions including macroelements (K, Na, Mg) and microelements (Li, B, Al, Si, V, Cr, Mn, Fe, Co, Ni, Cu, Zn, As, Se, Mo, Cd, Sn, Sb, Ba, Hg, Tl and Pb) using ICP-MS and SRμ-XRF technologies in chicken muscles in a state of Se deficiency. In chicken muscles, V, Cr, Mn, Cu, Cd, and Hg were significantly influenced by Se deficiency.

Levels of trace elements in chicken products and various parts of chicken samples have been widely reported in previously [22, 24]. Levels of trace elements contents in chicken samples are different in different chicken tissues, vary from the animal age [25], and involved in or related to the chicken health or the process of disease development [26, 27]. In the present study, the samples were collected from 25 days old chicken exhibiting typical signs of exudative diathesis. In this development stage, element distribution of 97.77% for macroelements, 2.20% for essential microelements and 0.031% for toxic microelements was observed. Among the detected macroelements, K (4343.77 ppm) was the top rank ion followed by Na (433.62 ppm), and Mg (353.48 ppm). Among the microelements, the essential microelement possessed 98.60%, but the potential toxic microelements only possessed 1.4%. In the present study, the top six microelements (**S2 Table**) were Si (100265.4 ppb), Fe (7307.22 ppb), Zn (6392.32 ppb), Al (1540.75 ppb), Cu (597.92 ppb), and B (292.89 ppb). This microelement distribution was significantly different from 90 day old chicken [27]. The top ranked microelement were Fe (183227 ppb), Si (131358 ppb), Zn (33968 ppb), Cu (5589 ppb), Mn (4890 ppb), Al (880 ppb), Se (706 ppb), and Pb (387 ppb) in the liver of 90 days old chicken, which are collectively higher than our observations [27]. In addition, Se levels (85.46 ppb) in chicken from control group was also lower than most of the microelements. Clinical symptom observed from our established models (data not shown) showed that chicken were more prone to get exudative diathesis between age of 20 days and 35 days (all birds showed typical symptoms of exudative diathesis in the present study), however, less or no chicken showed typical exudative diathesis or muscular dysfunction when chicken is above 50 days old. Moreover, chicken within 15 to 35 days old showed lower glutathione peroxidase (Gpx) activity but higher oxidative injury marker, MDA, and apoptosis genes, Bax and Caspase 3 than chicken from 55 days after Se deficiency treatment [7]. Except for the diet and age influencing factors, it may indicate the need and importance of sufficient microelements in chicken at this particular age, we also hypothesize that chicken at this stage (20–35 days old) are more sensitive to Se deficiency due to the lower body Se levels and selenoprotein expression and activity [7]. However, except the clinical observation more analysis such as specific genes expression or enzyme synthesis are needed to support our hypothesis.

In the present study, we analyzed 25 ions in Se deficiency muscles by ICP-MS and analyzed the elements distributions by SRμ-XRF. The results showed that the contents of V, Cr, Mn, Cu, Cd, and Hg were significantly increased; however, other elements were not influenced. This response indicated that there may be antagonistic effect between Se and these elements, which was consistent with previous reports in some organs or species. Se deficiency elevated Cu content without influencing Zn and Fe levels in rat liver [19], increased Zn and Cu levels in testis [28]. However, there were also some different voice about the effect of Se and these elements, in which Se deficiency did not influence the Hg concentration in fetal brain of mice [29], Se deficiency decreased Zn content but had no effect on Cu, Fe and Mn in mice plasma, and decreased Cu and Mn and increased Zn in kidney [28]. Thus, types of elements preserve altered responses to various levels of Se, also showed different responses in different tissues. Therefore, Se plays an important role in regulating ions profile in different organism or tissues. To confirm and identify the effect of Se deficiency on elements, we further detected the elements distribution in muscles sections. However, due to low sensitivity and low traces, some elements such as Se was not detected by SRμ-XRF technology. These findings support the idea that Se deficiency influences the elements distribution in muscle sections. However, the changing distribution images of elements showed different patterns. Ba, Co, Cu, Fe and V showed generalized accumulation while Cr, Mn, and Zn showed accumulation at various small points in muscles sections. We do not know why this different distribution happened for different elements, but in our previous study we found that Se deficiency decreased Ca levels in chicken muscles, and induced generally reduction of Ca in muscles section detected by SRμ-XRF method. This generally reduction of Ca in muscle was closely related to the expression levels of Ca channels and selenoproteins, and redox state of muscles [7]. In addition, Fe, Mn, Cu, and Zn are important components of enzyme, such as catalase (CATs), Cu, Zn-superoxide dismutase (SOD), and Mn-SOD, and the levels of these elements and the activities of these enzymes were also influenced by Se in different rat tissues, thus the levels of the elements also related to the status of redox, and the activities of these enzymes [28]. So the interaction between Se and other elements is not just synergistic or antagonistic effect, there are also some complicated modulating effect, such as gene modulation, transportation, Se compound forming, etc [18, 28]. So the different distribution patterns of Ba, Co, Cu, Fe, Cr, Mn, and Zn in muscles section may be correlated with these possible mechanisms. Complex correlation between Se and other elements were also observed through PCA. Se preserved positive correlation with B, Mg, Si, As and Sb, but negative correlations with other elements. In response to Se deficiency, these elements could be divided into several component groups and showed different correlation each other. Although there are some different microelements correlation between chicken muscles and rat liver [19], testis [28], mice brain [29], plasma, and kidney [28], and chicken liver [27]. These results showed the special microelements interaction in chicken muscles, and the difference between species and organs.

Among these affected ions, Cr, Cu, and Mn are the essential microelement for both animals and humans. Cu and Mn are the essential component of key enzymes such as glutamine synthetase, arginase, phosphoenolpyruvate decarboxylase, and mitochondrial superoxide dismutase [30–32]. Imbalance in levels of Cr, Cu and Mn influence the activity of types of enzymes and the normal biological function of organisms. Previous studies showed that Cu and Cr were involved in the induction or defense of oxidative stress [33]. Moreover, Cu, Mn and Cr were related to types of diseases such as neurological disorders, diabetes, cardiovascular disease, disturbed glucose tolerance, and fasting hyperglycemia [32, 33]. In our previous study, we observed that Se deficiency induced the oxidative stress, influenced the activities of antioxidative enzymes such as Gpx, and SOD in chicken muscles [7]. Therefore, combining the ion profiles and the related molecular changes may help us focus on some specific pathway and ion in Se deficiency disease. This study further indicates the important role of ion profile during studying some disease in chicken.

In the present study, Se deficiency also significantly induced higher levels of microelements that belong to potential toxic elements such as Cd, and Hg. As indicated in previous studies, Se may serve as antagonist counteracting the toxicity of toxic metals such as Hg, Cd, As, Ag, Pb, and Cu in plants and animals [16, 34–36]. Se also protects against Cd in chicken cells or tissues as reported in our previous studies [37, 38]. Therefore, the data in the present study well supports the results of these reports. The accumulation of these toxic microelements such as Cd, Pb, Mn, Hg, etc, may induce types of injuries in different tissues, such as the kidney dysfunction, cardiovascular disease, and muscular weakness [22, 39]. However, the concentration of Cd and Hg in the Se deficiency chicken muscles is just 0.52 ppb and 1.11 ppb that is much lower than the toxic concentration [40–43]. So the abnormal increase of the toxic element may not contribute to the muscular injuries induced by Se deficiency. However, the increased contents of these ions and accumulation in muscle sections showed that the Se deficiency may influence the transportation or discharge of these microelements as indicated in previous studies. This finding may provide some idea to further investigate the toxic or non-toxic mechanisms of these elements, as observed previously [17, 18, 36].

In summary, in the present study we analyzed 25 ions profile in chicken muscles. The ions profile in Se deficiency chicken showed its characteristic that has a different microelement levels and especially Se level. The lower Se levels combining with lower expression of selenoprotein may explain why chicken in this stage was more sensitive to Se deficiency. Se deficiency affected the contents of essential microelements (Cu, Cr, V and Mn), and potential toxic elements (Cd and Hg) in chicken muscles. In addition, difference in visual distribution of ions was generally consistent with the contents detected by ICP-MS, suggesting that the transportation or discharge of these microelements may be different in response to Se deficiency. PCA analysis also suggests the existence of complex correlation between different elements. Based on these findings It was elicited that Se may plays important and complex role in regulating ion profiles in chicken muscles.

## Supporting information

S1 Table Instrumental parameters for the ICP-MS. (DOCX)

S2 Table Elements content in chicken muscles. (DOCX)
