# Supplementary material for: Selenium deficiency-induced alterations in ion profiles in chicken muscle
Source: PLoS One. 2017 Sep 6;12(9):e0184186. doi: 10.1371/journal.pone.0184186 (PMC5587317; doi:10.1371/journal.pone.0184186)
Supplement: S1 Table — (DOCX) [file pone.0184186.s001.docx]

**S1 Table. Instrumental parameters for the ICP-MS**

| Items | Parameters |
| --- | --- |
| Frequency (MHz) | 27.12 |
| Reflect power (kW) | 1.55 |
| Sampling depth (mm) | 5.0 |
| Torch-H (mm) | 0.01 |
| Torch-V (mm) | -0.39 |
| Carrier gas (L/min) | 1.05 |
| Nebuliser pump (rpm) | 40 |
| S/C temperature (°C) | 2.7 |
| Oxide ions (156/140) | <2.0% |
| Doubly charged (70/140) | <3.0% |
| Nebuliser type | Concentric |
