# Supplementary material for: Selenium deficiency-induced alterations in ion profiles in chicken muscle
Source: PLoS One. 2017 Sep 6;12(9):e0184186. doi: 10.1371/journal.pone.0184186 (PMC5587317; doi:10.1371/journal.pone.0184186)
Supplement: S2 Table — (DOCX) [file pone.0184186.s002.docx]

**S2 Table. Elements content in chicken muscles**

|  |  | Macroelements | | | Essential microelements | | | | | | | | | | | | | | | Toxic microelements | | | | | | |
| --- | --- | --- | --- | --- | --- | --- | --- | --- | --- | --- | --- | --- | --- | --- | --- | --- | --- | --- | --- | --- | --- | --- | --- | --- | --- | --- |
|  |  | Na | Mg | K | B | Ba | Co | Cr | Cu | Fe | Mn | Mo | Ni | Sb | Se | Si | Tl | V | Zn | Al | As | Cd | Hg | Li | Pb | Sn |
| Con | Mean | 433613.90 | 353479.20 | 4343764.73 | 292.89 | 37.93 | 11.12 | 147.02 | 597.92 | 7307.22 | 268.07 | 7.26 | 24.95 | 11.60 | 85.46 | 100265.38 | 3.25 | 8.92 | 6392.32 | 1540.75 | 22.51 | 0.25 | 0.72 | 22.03 | 40.52 | 13.36 |
|  | SD | 31841.21 | 10721.32 | 120385.33 | 40.87 | 8.61 | 2.34 | 34.95 | 70.99 | 1305.49 | 26.66 | 0.75 | 3.90 | 2.71 | 12.33 | 25354.47 | 0.74 | 1.23 | 400.27 | 272.36 | 6.87 | 0.14 | 0.20 | 2.73 | 21.62 | 0.99 |
| -Se | Mean | 432041.45 | 352403.18 | 4407985.47 | 288.56 | 45.32 | 13.70 | 366.48 | 723.49 | 7846.08 | 472.39 | 7.86 | 29.70 | 11.31 | 27.90 | 104482.77 | 3.64 | 13.36 | 6353.98 | 1557.54 | 22.49 | 0.52 | 1.11 | 25.47 | 56.28 | 13.75 |
|  | SD | 26037.47 | 11440.49 | 151081.30 | 54.44 | 21.55 | 5.62 | 103.26 | 103.35 | 1256.20 | 154.25 | 0.20 | 15.32 | 1.27 | 3.53 | 24696.87 | 0.21 | 2.95 | 447.91 | 211.10 | 4.21 | 0.17 | 0.14 | 5.36 | 13.08 | 0.80 |
